# CardiOvaScular Mechanisms In Covid-19: methodology of a prospective observational multimodality imaging study (COSMIC-19 study)

**DOI:** 10.1186/s12872-021-02027-0

**Published:** 2021-05-08

**Authors:** Shirjel R. Alam, Anoop S. V. Shah, Kevin O. Ombati, Edward Nganga, Samuel Gitau, Khalid Makhdomi, Michael H. Chung, Sudhir Vinayak

**Affiliations:** 1 Manchester University, Manchester, UK; 2 London School of Hygiene and Tropical Medicine, London, UK; 3 Aga Khan University Hospital, Nairobi, Kenya; 4 Emory University, Atlanta, USA

**Keywords:** COVID-19, CMR, FDG, 18F-FDG-PET/CT, CTCA, Cardiovascular, Troponin, Cardiac injury, Imaging

## Abstract

**Background:**

8–28% of patients infected with COVID-19 have evidence of cardiac injury, and this is associated with an adverse prognosis. The cardiovascular mechanisms of injury are poorly understood and speculative. We aim to use multimodality cardiac imaging including cardiac magnetic resonance (CMR) imaging, computed tomography coronary angiography (CTCA) and positron emission tomography with 2-deoxy-2-[fluorine-18]fluoro-d-glucose integrated with computed tomography (18F-FDG-PET/CT) to identify the cardiac pathophysiological mechanisms related to COVID-19 infections.

**Methods:**

This is a single-centre exploratory observational study aiming to recruit 50 patients with COVID-19 infection who will undergo cardiac biomarker sampling. Of these, 30 patients will undergo combined CTCA and 18F-FDG-PET/CT, followed by CMR. Prevalence of obstructive and non-obstructive atherosclerotic coronary disease will be assessed using CTCA. CMR will be used to identify and characterise myocardial disease including presence of cardiac dysfunction, myocardial fibrosis, myocardial oedema and myocardial infarction. 18F-FDG-PET/CT will identify vascular and cardiac inflammation. Primary endpoint will be the presence of cardiovascular pathology and the association with troponin levels.

**Discussion:**

The results of the study will identify the presence and modality of cardiac injury associated COVID-19 infection, and the utility of multi-modality imaging in diagnosing such injury. This will further inform clinical decision making during the pandemic.

*Trial Registration*: This study has been retrospectively registered at the ISRCTN registry (ID ISRCTN12154994) on 14th August 2020. Accessible at https://www.isrctn.com/ISRCTN12154994

## Background

The number of people diagnosed with the coronavirus disease 2019 (COVID-19) [1] has surpassed 150 million worldwide claiming over 3.2 million deaths [2]. Patients with underlying cardiovascular comorbidity are more severely affected, with 8–28% of patients showing evidence of heart injury [3, 4]. These patients exhibit a substantially higher death rate [5]. The odds of in-hospital death were 80-fold higher in patients with elevated cardiac troponin concentrations [6].

At present, the cardiovascular pathology of injury in COVID-19 patients remain poorly understood, with the underlying mechanism being speculative [6, 7]. To date, cardiac manifestations of COVID-19 infection have been limited to case reports and series or in patient populations who have already survived COVID-19 with imaging evaluating medium-term cardiac sequelea of COVID-19 [8–10].

Using cardiac magnetic resonance (CMR) imaging [11–13], computed tomography coronary angiogram (CTCA) [14] and positron emission tomography with 2-deoxy-2-[fluorine-18]fluoro-D-glucose integrated with computed tomography (18F-FDG-PET/CT) [15] during acute COVID-19 infection, we intend to investigate structural and functional changes of the myocardium and arterial vasculature, including evidence of inflammation, fibrosis and prevalence of underlying coronary atherosclerosis.

Our principal aims are to identify the cardiac pathophysiological mechanisms related to COVID-19 infection that would influence care and clinical outcomes and provide insight into potential therapeutic targets.

## Methods/design

This is a single-centre exploratory observational study involving participants diagnosed with COVID-19 receiving care at the Aga Khan University Hospital in Nairobi, Kenya. We aim to recruit around 50 patients with COVID-19 infection with 20 to 30 patients undergoing cardiac imaging. If feasible we will aim to image 15–20 with plasma troponin levels, using a high-sensitivity assay, above the 99th centile upper reference limit (URL) and 5–10 patients with troponin levels within the normal reference range. We will additionally identify a control population of individuals where COVID-19 is suspected but excluded following testing. We will attempt to match controls by age and sex to the COVID-19 positive population. All patients not requiring non-invasive and invasive ventilation will be eligible for recruitment.

### Selection criteria

Inclusion criteria for patients diagnosed with COVID-19 include those over the age of 18 years, testing positive on PCR testing and ideally within 2 weeks of a positive test. Patients will be excluded based on the following conditions: (1) prior diagnosis of myocardial infarction, coronary revascularisation or cardiac surgery; (2) requiring invasive or non-invasive ventilation; (3) inability to undergo CT or CMR scanning; (4) severe renal failure (estimated glomerular filtration rate < 30 mL/min); (5) Major allergy to iodinated contrast media/gadolinium; (6) pregnancy or breast feeding; (7) inability to give informed consent; (8) contraindication to imaging example metal fragments in the eye. Similarly exclusion criteria will apply to patients over the age of 18 years chosen as controls and testing negative for COVID-19.

We will also explore stratification by clinical parameters of COVID-19 severity. As such we will also provide baseline characteritics and imaging parameters by clinical requirement of oxygen supplementation.

### Protection of staff and patients from COVID-19 infection

All staff involved in scanning patients will wear personal protective equipment (PPE) in line with hospital policy, and follow protocols created by the study team including physicians from the infectious diseases team. Patients will be scanned last on the list followed by a deep clean to the scanning area to prevent nosocomial cross infection. Routine clinical scans will be prioritised over research scans. No aerosol producing procedures will be performed.

### Image acquisition

At initial recruitment, patients will undergo blood sampling for high-sensitivity cardiac troponin, N-terminal pro B-type natriuretic peptide and high-sensitivity C Reactive protein. A subset of patients will be selected for multimodality cardiovascular imaging. These participants will undergo two visits for a combined CTCA and 18F-FDG-PET/CT as soon as possible, followed by a CMR on another visit. All CTCA and 18F-FDG-PET/CT will be performed within 2 weeks of presentation to maximise sensitivity of the scan. All efforts will be made to ensure CMR scans are also performed within 2 week, however scanning beyond this point will be performed if it is logistically impossible to perform the scan within this window.

#### Computed tomography coronary angiogram

CTCA scanning will be ECG-gated and performed in diastole during a single breath hold with prospective electrocardiographic gating, detector collimation 64 × 0.625 mm, tube voltage 120 kV and window of acquisition 70–90% (or wider if necessary due to heart rate). Tube current will vary depending on BMI using a pre-specified manufacturer protocol. After the acquisition of scout images, CTCA will be performed with iodinated contrast (Ultravist 370 mg/mL) in a biphasic injection protocol. Image acquisition will be triggered by contrast enhancement of 100 HU in the ascending aorta. In order to optimise the quality of the CTCA scan images, heart rate control will be pharmacologically achieved if appropriate, and in addition sublingual glyceryl trinitrate will be administered.

#### 18F-FDG-PET/CT

Participants will undergo 18F-FDG-PET/CT imaging after high-fat, low-carbohydrate meal for 24 h with 18 h fast to reduce physiologic myocardial FDG uptake [15, 16]. The PET imaging will be performed 60–90 min after administration of 10–15 mCi of 18F-FDG using a GE Discovery MI series PET/CT (64 slice) scanner. The carotid arteries will be the superior aspect of imaging, and the entire thoracic aorta will be covered using 3 min different bed positions with additional dedicated 10 min cardiac acquisition. CT images will be obtained immediately after PET scan acquisition and the images co-registered following re-construction.

#### Cardiac magnetic resonance

Standard CMR images will be acquired as previous [17–19]. In brief, standard pilot images and cardiac views will be obtained using breath-held acquisitions. Sequences will be as follows: (1) HASTE/White blood axial; (2) cines 2, 3 and 4 chamber; (3) T1 map short axis basal and mid-ventricular short axis slices, with additional long axis images if possible; (4) T2 map short axis basal and mid-ventricular short axis slices, with additional long axis images if possible; (5) post gadolinium contrast PSIR short axis cine stack, 2, 3 and 4 chamber; (6) post contrast T1 map short axis basal and mid-ventricular short axis slices; (7) aortic flow map.

Gadolinium-based contrast agent will be intravenously administered. Myocardial infarction and fibrosis will be identified by the presence of hyperenhancement in the myocardium in late enhancement images, acquired using a T1-inversion sequence with inversion time optimised on a patient-by-patient basis [20]. Multiple slices will be acquired in the short axis view with inversion time further optimised for each slice. Two- and 4-chamber late enhancement views will also be acquired to verify presence or absence of late enhancement. Late enhancement images will be acquired approximately 5–12 min after gadolinium infusion. T1 characteristics, extra cellular volume as well as the presence of cardiac oedema by T2-maps will be assessed [11, 13, 21].

### Image analysis

All scans will be anonymised, with analysts blinded to all blood results and other scanning modalities. The cardiac MRIs will be analysed in Manchester (UK), PET scans in Bristol (UK) and CTCAs in Edinburgh (UK). There will be no discussion regarding scans between the centres. When a second opinion is needed, it will be sought from an independent radiologist in Nairobi (Kenya). All analysis will be performed by expert cardiologists trained in the relevant cardiac imaging modalities. The second opinion, when sought, will be performed by expert cardiothoracic radiologists.

#### Computed tomography coronary angiography

The presence of coronary artery disease in each major coronary artery, and the main side branches will be documented based on degree of stenosis. In addition presence of coronary calcification, other cardiac or vascular pathology, pericardial pathology and aortic/main pulmonary artery diameter will be recorded.

#### 18F-FDG-PET/CT

Arterial inflammation: Aortic inflammation will be assessed as previous [15, 22]. In brief CT and PET scan images will be co-registered automatically. The aorta will be divided into axial slices, and the tracer standardised uptake value (SUV) measured from a point distal to the origin of coronary vessels to avoid myocardial spill-over. Regions of interest will be drawn around the aorta in the axial position, repeated along the length of the aorta. A mean arterial SUV will be derived from the average of the maximum SUV values in serial axial measurements. Similarly the average of SUV measurements from the  venous pool will derive the mean venous background SUV. The target-to-background (TBR) ratio will then be calculated by dividing the mean arterial SUV by the mean venous SUV.

Myocardial inflammation: Myocardial uptake will be assessed as previous [23–27]. In brief CT and PET scan images will be co-registered. Analysis will be performed on short-axis cardiac slices for later comparison with CMR using 17 segment model [28]. Liver SUV uptake will be measured by drawing a hepatic region of interest. Uptake in the heart as a whole will be scored according to a visual scale to assess cardiac suppression of FGD uptake (ie adequate fasting/dietary preparation).

Visual assessment of myocarditis:

A visual assessment of FDG uptake (on diagnostic scans) that signifies myocarditis will be categorised: None—1, focal on diffuse—2, Focal—3, Diffuse—4.

Objective assessment of myocarditis:

Each segment of the 17 segment model, will be assessed for myocardial uptake visually. In addition, regions of interest within each myocardial segment of the 17-segment model will be drawn avoiding the blood pool and liver.

A mean cardiac SUV will be derived from the average of the maximum SUV values in serial short axis measurements (Sum of each segment /17).

Assessment of each segment of the 17 segment model will be performed in 3 different ways as follows.

1. Raw SUV uptake compared to blood pool uptake will be calculated for each segment, and categorised.
2. TBR (target-to-background-ratio) will be calculated by dividing each cardiac segment SUV by the mean blood pool SUV
3. Each segment indexed to the mean blood pool will also be categorised (0—uptake < mean blood pool uptake),1—uptake = mean blood pool uptake,2—uptake > mean blood pool uptake but < liver uptake, 3—(uptake > liver uptake)

#### Cardiac magnetic resonance imaging

Ejection fraction, regional wall motion abnormalities, myocardial fibrosis, oedema and presence of infarction will be determined as previously described [11, 13, 29]. Briefly, epicardial and endocardial contours will be drawn around diastolic phase cines, and endocardial contours around systolic phase cines to calculate cardiac volumes and myocardial mass. The myocardium will be separated into 16 segments of the American Heart Association 17-segment model excluding the apex [28]. T1 values,T2 values, extra cellular volume and the presence of late gadolinium enhancement will be generated for each segment using dedicated software. T1 values will indicate fibrosis, T2 values oedema and gadolinium enhancement the presence of infarction as well as fibrosis depending on distribution [11, 13, 20].

## Primary objectives

We have four primary objectives:

- Identify the proportion of patients with biochemical evidence of myocardial injury or strain with the presence of elevated cardiac troponin.
- Identify significant atherosclerotic disease potentially causing cardiac injury with the presence of obstructive coronary artery disease on CTCA (> 50%).
- Identify myocardial disease using CMR by determining ejection fraction, presence of myocardial infarction (sub-endocarditis gadolinium enhancement), myocarditis (mid-wall/epicardial gadolinium enhancement) or myocardial oedema (elevated T1 values and T2 values)
- Identify vascular inflammation by 18F-FDG-PET/CT by quantifying increased FDG uptake in aorta. Determining the presence of different patterns of FDG uptake in myocardium will be a secondary exploratory outcome.

## Sample size calculation and statistics

Based on published literature we based our sample size calculation on the assumption that 1 in 4 of hospitalised COVID-19 patients will have elevated cardiac troponin [3]. In sample size calculations, we estimated that with 50 patients, we would be able to estimate the confidence interval for a prevalence of 25% with lower and upper intervals of 14.1% and 37.4% respectively, and that we would have 80% power for an alpha of 0·05 to test the null hypothesis that the prevalence was < 10%. This is an exploratory observational cross-sectional study to investigate the potential cardiac mechanisms in patients with COVID-19. As such we have not performed any formal power calculations based on imaging parameters.

Baseline clinical and imaging data will will be expressed as the mean ± standard deviation (SD) for parametric data and median (interquatile range) for non-parametric data. Categorical data will be presented as proportions. We will conduct hypothesis testing based on imaging parameters comparing (a) controls and patients and (b) within patients, those stratified by troponin concentrations. For within patient analysis we will attempt to compare individuals stratified above and below the 99th centile for cardiac troponin concentration. If the number of patients above the 99th centile for cardiac troponin are small, we will group patients by equal strata of cardiac troponin value. We will also explore stratification by clinical parameters of COVID-19 severity. As such we will provide also provide baseline characteritics and imaging parameters by clinical requirement of oxygen supplementation. *P* value of less than 0.05 will indicate statistical significance.

## Discussion

In order to understand cardiac complications secondary to COVID-19, this study aims to utilise multimodality cardiac imaging to identify any morphological and functional cardiovascular changes associated with infection. To date, whilst data has shown that myocardial injury is prevalent in patients with COVID-19, cardiac imaging has either been limited to case reports and small case series, performed after recovery using single modalities or using study designs highly susceptible to selection/reporting [8–10].

To our knowledge, our study represents the first multi-modality cardiac imaging study, with each technique attempting to elucidate specific pathophysiological processes. CTCA will examine the presence of coronary disease associated directly or indirectly with elevated cardiac troponin concentrations, and its absence would imply Type 2 myocardial infarction in the correct context. CMR will evaluate cardiac function and dysfunction including structural evidence of heart failure resulting from either ischaemic or non-ischaemic cardiomyopathy (e.g. stress cardiomyopathy). It will also identify myocardial infarction or fibrosis caused by myocarditis. 18F-FDG-PET/CT can assess for myocardial and vascular inflammation that would delineate if myocardial oedema (seen on CMR) is caused by direct cardiac injury or as part of a generalised inflammatory process.

Identification of each pathophysiological process will inform future studies in evaluating optimal choices of therapeutic agents; (1) anti-platelet and anti-coagulation agents for atherosclerotic disease or thrombotic occlusions; (2) Blood pressure and oxygen support in the presence of type 2 myocardial infarction; (3) anti-viral or anti-inflammatory strategies for a primary myocarditic processes; (4) early and long term cardioprotective therapy for myocardial dysfunction.

There are several limitations and considerations that need to be taken into account with regards to our study design. First, given the imaging strategy undertaken, patients must be fit enough for example to breathhold. As such our study primarily aims to recruit COVID-19 cases who are not requiring invasive or non-invasive ventilation. Our findings may therefore not be generalisable to severe cases of COVID-19. Second, recent evidence has shown the role of anti inflammatories in managing patients with COVID-19. These drugs are likely to modulate the degree of inflammatory signal seen in the myocardium and arterial tree and may underestimate the true pathophysiological effect of the disease on the heart. Thirdly, manual selection or regions of interest during scan analysis is liable to error. This may be compounded by diminished scan quality due to infected patients being short of breath or tachycardic. Finally, the observational trial aims to recruit a relatively small number of patients, and clinical outcomes will not be evaluated. However there is a lack of data and well designed studies investigating COVID-19 cardiovascular injury, and the study should provide valuable insights.

## Data Availability

Data sharing is not applicable to this article as no datasets were generated or analysed during the current study.

## Abbreviations

CMR: Cardiac magnetic resonance
CTCA: Computed tomography coronary angiography
FDG-PET: ^1^^8^F-fluorodeoxyglucose positron emission tomography
ROI: Regions of interest
SUV: Standardized uptake value
TBR: Target-to-background ratio
